# Emerging Signaling Pathway in Arcuate Feeding-Related Neurons: Role of the Acbd7

**DOI:** 10.3389/fnins.2017.00328

**Published:** 2017-06-23

**Authors:** Damien Lanfray, Denis Richard

**Affiliations:** Centre de Recherche de l'Institut Universitaire de Cardiologie et de Pneumologie de Québec, Université LavalQuébec, QC, Canada

**Keywords:** hypothalamus, leptin, POMC, ACBD7, food intake behavior

## Abstract

The understanding of the mechanisms whereby energy balance is regulated is essential to the unraveling of the pathophysiology of obesity. In the last three decades, focus was put on the metabolic role played by the hypothalamic neurons expressing proopiomelanocortin (POMC) and cocaine and amphetamine regulated transcript (CART) and the neurons co-localizing agouti-related peptide (AgRP), neuropeptide Y (NPY), and gamma-aminobutyric acid (GABA). These neurons are part of the leptin-melanocortin pathway, whose role is key in energy balance regulation. More recently, the metabolic involvement of further hypothalamic uncharacterized neuron populations has been suggested. In this review, we discuss the potential homeostatic implication of hypothalamic GABAergic neurons that produce Acyl-Coa-binding domain containing protein 7 (ACBD7), precursor of the nonadecaneuropeptide (NDN), which has recently been characterized as a potent anorexigenic neuropeptide capable of relaying the leptin anorectic/thermogenic effect via the melanocortin system.

## Introduction

According to the World Health Organization (http://www.who.int/mediacentre/factsheets/fs311/en/) worldwide obesity prevalence has more than doubled since 1980. This situation is alarming given that obesity is often associated with costly diseases that include type-2 diabetes and cardiovascular diseases. In such a context, it appears urgent to improve the strategies to prevent or treat obesity, which cannot be elaborated without a deep understanding of the pathophysiology of excess fat deposition, hence the mechanisms whereby energy balance is regulated.

Obesity translates an imbalance between energy intake and energy expenditure leading to fat accumulation. In that respect, study of the causes of excess eating represents an inescapable step to understand obesity. Food intake as well as and energy expenditure are controlled by complex brain networks involving (i) cortical executive circuits, responsible for the self-control of eating and physical activity, (ii) corticolimbic reward pathways, which are involved in the integrations of hedonic and motivational signals, and (iii) autonomic hypothalamic and brainstem circuits that modulate the activity of the executive and reward structures while integrating peripheral homeostatic signals and controlling energy expenditure components (Gautron et al., 2015; Richard, 2015; Caron and Richard, 2017). Neurons of those networks produce numerous receptor types, neuropeptides and neurotransmitters that have been grouped into “pro-anabolic” (promoting obesity) or “pro-catabolic” (preventing obesity) chemical mediators.

## Autonomic regulation of energy homeostasis

The autonomic circuits regulating energy balance mainly consist in two brain structures, namely the hypothalamus and brainstem, which coordinate their respective activity to control energy intake, by modulating the rostral forebrain appetite network (executive and reward systems), and to control energy expenditure, by for instance modulating brown adipose tissue (BAT) non-shivering thermogenesis. Due to their anatomic locations, hypothalamic and brainstem neurons of those two regions are able of sensing homeostatic hormones and nutrients translating energy balance fluctuations. Notably, the hypothalamus and brainstem are located near to circumventricular organs (CVOs), which are devoid of blood brain barrier allowing direct contact with nutrients and hormones (Schwartz et al., 2000).

The hypothalamus is constituted of nuclei comprising neurons involved in autonomic functions including the control of energy intake and energy expenditure (Gautron et al., 2015; Richard, 2015; Caron and Richard, 2017). Those neurons, which are still to be fully characterized are located in nuclei including the arcuate nucleus (ARC), ventromedial nucleus (VMH), dorsomedial hypothalamic nucleus (DMH), lateral hypothalamus (LH), preoptic area (POA), and paraventricular hypothalamic nucleus (PVH) (Schwartz et al., 2000). They for instance convey homeostatic signals between the hypothalamus and appetitive rostral forebrain systems (Gautron et al., 2015; Richard, 2015; Caron and Richard, 2017). The brainstem comprises the dorsal vagal complex (DVC), which includes interconnected neurons found in the nucleus of the solitary tract (NTS), area postrema (AP), and dorsal motor nucleus of the vagus nerve (Blevins and Baskin, 2010; Schwartz, 2010; Simpson and Bloom, 2010). Other brainstem structures including the pontine parabrachial nucleus (PBN), raphe pallidus (RPa), periaqueductal gray (PAG), and lateral paragigantocellular nucleus have been associated with SNS-mediated non-shivering thermogenesis, by mainly conveying information from the hypothalamus to the interscapular brown adipose tissue (iBAT) (Morrison and Nakamura, 2011).

### The ARC in energy homeostasis

The hypothalamic nucleus that has been the most investigated in recent years with regard to energy homeostasis is undoubtedly the ARC (Gautron et al., 2015; Richard, 2015; Caron and Richard, 2017). The ARC is ventrally located on each side of the third ventricle just above the median eminence, a CVO allowing penetrance of peripheral hormones and nutrients (Schwartz et al., 2000; Richard, 2015). The ARC contains neurons producing proopiomelanocortin (POMC) and cocaine and amphetamine regulated transcript (CART) as well as neurons co-localizing agouti-related peptide (AgRP), neuropeptide Y (NPY), and gamma-aminobutyric acid (GABA), whose role in energy balance regulation have been acknowledged for years (Gropp et al., 2005; Luquet et al., 2005; Mayer and Belsham, 2009; Krashes et al., 2013). The NPY/AgRP/GABA- producing neurons exert anabolic effects while POMC/CART neurons are involved in catabolic processes. Those neurons have been referred to as “first order” neurons and project to “second order” neurons located in energy homeostasis-related nuclei, including the PVH and VMH, which individually form with the ARC prominent duets in the regulation of energy balance (Schwartz et al., 2000; Balthasar et al., 2005; Elmquist et al., 2005; Balthasar, 2006; Morton et al., 2006; Richard, 2015). Recently, single cell analysis performed in the hypothalamus revealed an important heterogeneity of ARC cells (Romanov et al., 2017), indicating that additional investigations will have to be make in order to fully characterized hypothalamic regulations of whole body homeostasis.

### The melanocortin system

ARC POMC/CART and NPY/AgRP/GABA neurons are major constituents of the melanocortin system, which is recognized as playing a genuine role in energy balance regulation (Adan et al., 2006; Butler, 2006; Cone, 2006; Ellacott and Cone, 2006; De Jonghe et al., 2011; Xu et al., 2011). POMC/CART neurons exert their hypophagic and thermogenic effects mainly by releasing the melanocortins α- and β-melanocyte-stimulating hormone (MSHs). α- and β-MSHs activate the melanocortin receptors 3 and 4 (MC3R, MC4R) to increase food intake and reduce energy expenditure (Cone, 2006). Interestingly, recent report indicate that POMC neurons are also able to released β-endorphin instead of MSHs indicating that those neurons should also act as orexigenic neurons (Koch et al., 2015). However, investigations performed on POMC knockout revealed morbid obesity resulting from hyperphagia as well as hypometabolism (Yaswen et al., 1999), indicating that POMC neurons mainly act as anorexigenic neurons. While the *Mc4r* knockout mice exhibit marked obesity, resulting from hyperphagia and hypometabolism (Huszar et al., 1997; Butler and Cone, 2003; Butler, 2006), genetic disruption of the *Mc3r* lead to a modest obesity phenotype (Chen et al., 2000; Butler and Cone, 2002, 2003), suggesting that MC4R constitutes the major MCR receptor involved in energy homeostasis. The prominent role of Mc4r in the hypothalamic regulation of energy balance has been confirmed in humans, in whom the *Mc4r* mutation leads to one of the most common forms of monogenic obesity (Coll et al., 2004).

NPY/AgRP/GABA neurons are also part of the melanocortin system. Together with inhibiting the activity of the POMC/CART neurons through a GABAergic effect (Pu et al., 1999), they release AgRP, a characterized anabolic peptide able to competitively inhibit α–MSH binding to the MC4R (Ollmann et al., 1997). AgRP has also recently been described as a biased agonist of MCR coupling to the inwardly rectifying potassium channel Kir7.1 (Ghamari-Langroudi et al., 2015). AgRP production is increased by fasting (Liu et al., 2012), supporting a physiological role for this peptide in the ARC control of energy homeostasis. Interestingly, non-conditional single KO AgRP^−/−^ as well as double KO AgRP^−/−^; NPY^−/−^ display normal energy homeostasis (Qian et al., 2002), suggesting that NPY/AgRP neurons are dispensable in the hypothalamic control of energy homeostasis. However, the post-natal genetic disruption of AgRP induces hypermetabolism and hypophagia (Luquet et al., 2005), demonstrating some physiological relevance of the neuron population. NPY has been considered as a robust orexigenic neuropeptide mainly by acting on Y1 and Y5 receptors (Richard, 2015). Interestingly, experiments performed in mice have indicated that the deletion of both Y1 and Y5 receptors induces anorexigenic effects (Nguyen et al., 2012), suggesting that Y1 and Y5 are receptors involved in energy balance. In contrast, the genetic disruption of *Npy* does not produce a lean phenotype or does not increase fasting-induced food intake, suggesting that *Npy* is not essential to the hypothalamic control of energy homeostasis. On the other hand, several investigations performed on *Npy* knockout mice revealed that those mice are less sensitive to high fat diet (Patel et al., 2006) (ref) and leptin (Erickson et al., 1996), which suggest that NPY could play a significant role in the hypothalamic integration of homeostatic signals. In that regard, future investigations appear require to further entirely decipher the role of NPY and its receptor in regulation of energy homeostasis.

### The ARC as a relay for peripheral homeostatic signals

As alluded to above, ARC cells, including POMC/CART and NPY/AgRP/GABA neurons, are strategically located to act as relays between peripheral homeostatic signals and other hypothalamic circuits involved in energy balance. The homeostatic signals can be anabolic and catabolic circulating hormones and nutrients.

Among all characterized anabolic hormones, ghrelin has emerged as one of the most significant ones. Ghrelin, which is largely produced by stomach cells under fasting (Sanchez et al., 2004a,b; Vallejo-Cremades et al., 2004), promotes food intake and decreases energy expenditure (Ueno et al., 2005) by acting on the growth hormone secretagogue receptor type 1A (GHS-R) (Kim et al., 2003; Egecioglu et al., 2006; Bresciani et al., 2008). The physiological role of ghrelin in the energy homeostasis is supported by investigations performed in mice revealing that genetic disruption of *Ghrelin* or *Ghs-r* can prevent high-fat induced obesity (Lee et al., 2016). GHS-R is widely produced in the central nervous system, where ghrelin exerts its effects in several brain regions (Zigman et al., 2006). Furthermore, experiments performed in GHS-R-deficient mice have shown that specific re-expression of the GHS-R in ARC AgRP neurons partially restores the orexigenic effect of the ghrelin (Wang et al., 2014), which confirms the involvement of AgRP-producing neurons as relays in the hypothalamic ghrelin signaling pathway.

Among all key homeostatic hormones, leptin, which is produced mainly by white adipose tissue (MacDougald et al., 1995; Cinti et al., 1997; Niijima, 1998) (WAT), is considered as one of the most prominent catabolic circulating hormones (Elmquist et al., 1997; Friedman and Halaas, 1998; Elias et al., 1999; Friedman, 1999; Gautron et al., 2010; Gautron and Elmquist, 2011). Leptin levels increase with fat mass (Lonnqvist et al., 1997). Its access to the brain is insured by an active transport system apparently involving tanycytes (Balland and Prevot, 2014; Balland et al., 2014). Leptin acts by activating the LepRb receptor, which can be found in several populations of ARC cells including POMC/CART and NPY/AgRP/GABA neurons. Genetic disruption of the gene encoding leptin (*ob*) or its receptor (*Lepr*) leads to marked obesity, hyperphagia and reduced BAT thermogenesis in mice (Thenen and Mayer, 1976; Leiter et al., 1983; Garris, 1987, 1989; Malik and Young, 1996; Mizuno et al., 1998; Garris and Garris, 2004; Goncalves et al., 2009).

Notably, the obesity induced by the disruption of leptin signaling resembles that observed following *Pomc* or *Mc4r* nullification (Trevaskis and Butler, 2005). In such context one may argue the presence of a functional link between leptin and the melanocortin system, all the more so that POMC/CART and NPY/AgRP/GABA neurons express *LepR* mRNA (Baskin et al., 1999; Elias et al., 1999; Wilson et al., 1999; Williams et al., 2010) and that leptin increases and decreases the mRNA levels of *Agrp* and *Pomc* respectively (Elias et al., 1999; Cowley et al., 2001; van den Top et al., 2004; Takahashi and Cone, 2005). However, experiments performed in mice revealed that mice lacking *Lepr* on POMC neurons (e.g., *Pomc*-Cre, *Lepr*^*lox*/*lox*^ mice) (Balthasar et al., 2004), on AgRP neurons (e.g., *Agrp*-Cre, *Lepr*^*lox*/*lox*^ mice) (Tong et al., 2008) and on both POMC and AgRP neurons (e.g., *Pomc*-Cre, *Agrp*-Cre, *Lepr*^*lox*/*lox*^ mice) (van de Wall et al., 2008) develop mild obesity, which suggests that the POMC/CART and NPY/AgRP/GABA could not be the only neurons interfacing the catabolic action of leptin. In that regard, the suggestion has been made that there could exist another population of neurons involved in the hypothalamic leptin signaling pathway. In that regard, mice lacking *Lepr* on GABA-producing neurons (Vong et al., 2011) (*Vgat*-Cre, *Lepr*^lox/lox^ mice) develop strong obesity. Apparently, there are LepR-expressing GABAergic neurons, distinct from NPY/AgRP neurons that exert an inhibitory tone onto POMC neurons, which could be blunted by leptin (Vong et al., 2011). This presumption has however been challenged by experiments performed in obese model mice homozygous for the *Lepr*^S1138^ allele, in which the ability to acutely decrease the GABA inhibitory tone is unaltered, despite the loss of the catabolic effects of leptin. In such a context, the characterization of the unidentified neurons capable of modulating POMC/CART neurons in response to leptin can be seen as major challenge of the current research in the neurobiology of obesity.

## ARC anorectic neurochemical candidates interfacing leptin and the melanocortin systems

The list of ARC neuromediators other than those released by POMC/CART and NPY/AgRP/GABA neurons, which could relay the catabolic message of leptin via the melanocrotin system, is rather short. It includes prolactin-releasing peptide (PrRP), neurotensin, diazepam-binding inhibitor/acylcoA binding protein (DBI/ACBP) and acyl-coA-binding domain containing protein 7 (ACBD 7).

PrRP is a potent anorexigenic neuropeptide acting via the Neuropeptide FF receptor 2 (NPFF2) receptor (Engstrom et al., 2003). It is expressed by ARC neurons harboring the LEPRs (Ellacott et al., 2002). Its expression is reduced in leptin-resistant Zucker rats, suggesting that leptin can directly activate PrRP neurons (Ellacott et al., 2002). Moreover, a recent report revealed that PrRP was strongly enriched in LEPRs-positive neurons (Allison et al., 2015), indicating that PrRP positive neurons should play significant role in the leptin signaling pathway. However, mice lacking PrRP in LEPRs producing neurons (*PrRP*-Cre, and *Lepr*^flox/flox^ mice) only develop mild obesity mainly due to lower energy expenditure (Dodd et al., 2014), which suggests that PrRP-producing neurons do not represent a major relay between leptin and the melanocortin signaling pathway.

Neurotensin is a 13-amino acid neuropeptide produced in the ARC, PVN, and DMH (Jennes et al., 1982; Beck et al., 1998). Its injection into the PVN decreases food intake (Stanley et al., 1983) and its production is increased by leptin injection (Beck et al., 1998). However, the evidence that leptin could mainly act through the ARC neurotensin-containing neurons is weak. Indeed, mice lacking the LEPR on NT neurons (*Nt*-Cre, *Lepr*^lox/lox^ mice) develop only mild obesity (Leinninger et al., 2011).

The diazepam-binding inhibitor/AcylCoA binding Protein (DBI/ACBP) is a 87/88 amino acid polypeptide produced by astroglial cells in the rodent hypothalamus (Tonon et al., 1990). DBI/ACBP is processed into several gliopeptides, including the octadecaneuropeptide (ODN), a potent anorexigenic peptide (de Mateos-Verchere et al., 2001; do Rego et al., 2007; Lanfray et al., 2013, 2016). Although ODN is exclusively produced by astroglial cells in the hypothalamus (Tonon et al., 1990), it can be considered as a candidate in the leptin-melanocortin pathway since astrocytes have been shown to produce LEPRs (Hsuchou et al., 2009a,b). Interestingly experiments perform in laboratory rodents indicate that the anorexigenic effect of icv injection of ODN is relayed by central activation of the MC3/4R (Lanfray et al., 2013), suggesting that ODN could directly activate POMC/CART neurons. Moreover, it has been recently demonstrated that the pharmacological disruption of the endozepine metabotropic receptor blunts the anorexigenic effect of the leptin (Lanfray et al., 2016), indicating that ODN may be involved in the leptin-melanocortin pathway. However, it has been demonstrated that the *Dbi/Acbp* mRNA levels are not affected by leptin in mice (Compere et al., 2010), which suggests that ODN is probably not a major component in the action of leptin on the melanocortin system.

## Acyl-CoA-binding domain containing protein 7 (ACBD7) as a neuromediator involved in the central effects of leptin

### ACDB7 origin

ACBD7 is a member of the ACBD protein family, which includes proteins containing the acyl-coA-binding domain motif signature (Burton et al., 2005; Neess et al., 2015). This protein family contains the well-characterized ACBD1, also known as DBI/ACBP (see above), which is known to be involved in numerous intracellular processes including fatty acid, glycerolipid, and glycerophospholipid biosynthesis, cellular differentiation and proliferation, and β-oxydation. Several hypothetical related-proteins have been characterized *in silico*, including ACBD7, which represents the product of a well-conserved paralog gene of the DBI/ACBP. Interestingly, sequence analysis has revealed that ACBD7 contains all the residues relevant for DBI/ACBP stability and acyl-CoA binding efficiency (Burton et al., 2005). However, while the three-dimensional conformation of the ACBD7 has been characterized (Neess et al., 2015), its ability to bind acyl-CoA esters remains to be established.

Considering the highly conserved exon/intron structure (Lanfray et al., 2016) of Acbd7, it has been postulated that the duplication of the ancestor gene occurs prior to the divergence of fish and higher vertebrates (450 Mya) (Burton et al., 2005). Interestingly, as for its paralog gene product (i.e., Dbi/Acbp), ACBD7 contains strongly conserved lysine allowing for the production of potential bioactive peptides, including a potential bioactive central fragment, released from a tryptic maturation process. By using a mass spectrometry-multiple reaction monitoring MS-MRM approach, we demonstrated that a 19-amino acid peptide-derived from ACBD7 (called nonadecaneuropeptide—NDN) was present in the mouse hypothalamus, demonstrating that Acbd7 was produced and processed *in vivo* (Lanfray et al., 2016).

The expression of Acbd7 in the hypothalamus suggests that ACBD7 may exert specific autonomic functions (Neess et al., 2015). We recently confirmed that Acbd7 was expressed by ARC and PVN neurons (Lanfray et al., 2016), two structures described above as key in the hypothalamic regulation of energy balance. Additionally, immunohistochemistry experiments have shown that ACBD7 is produced by ARC neuronal cells apparently differing from NPY/AgRP/GABA and POMC/CART (Lanfray et al., 2016). Additionally, our investigation indicates that ACBD7 immunoreactivity is co-localized with VGAT immuno-labeling (Lanfray et al., 2016), demonstrating that ACBD7 is produced by GABAergic neurons in the hypothalamus.

### Effects of ACBD7 on energy homeostasis

The observation that ARC ACBD7 produced a fragment homologous to the anorexigenic DBI/ACBP-derived peptide ODN prompted us to hypothesize that ACBD7-containing neurons could be involved in energy balance, all the more so that there existed a *Acbd7* polymorphism that had been associated to obesity in humans (Comuzzie et al., 2012).

To determine the role of ACBD7 on energy homeostasis, we assessed the effects of NDN on both food intake and energy expenditure. Our investigations performed in mice revealed that intracerebroventricular (icv) administration of NDN induced an early and marked inhibition of food intake in fasted mice (Lanfray et al., 2016). Our investigations also demonstrated that the icv injection of NDN increased both O_2_ consumption and UCP-1 expression in interscapular BAT (Lanfray et al., 2016), suggesting that NDN could also enhance energy expenditure. We also observed that the subchronic treatment with NDN (5 days) reduced food efficiency. Notably, the anorexigenic effect of NDN was blunted by the antagonism of the MC4R (Lanfray et al., 2016), the main effector of the melanocortin signaling pathway, suggesting that NDN acted upstream to the melanocortin signaling pathway.

We also demonstrated that Acbd7 mRNA levels, the ACBD7 protein levels and hypothalamic NDN levels varied with energy availability (Lanfray et al., 2016). This supports the notion that ACBD7-producing neurons are stimulated by one or several catabolic hormones/factors. In that regard, we demonstrated that the leptin treatment could increase the production of both ACBD7 and NDN, suggesting that leptin is able to stimulate ACBD7-producing neurons (Lanfray et al., 2016). Moreover, we demonstrated that the acute pharmacological disruption of the endozepines metabotropic receptor signaling blunted the anorexigenic effect of leptin (Lanfray et al., 2016).

Altogether the data accumulated so far suggest that (i) ACBD7 and NDN are produced by ARC GABAergic neurons different from POMC/CART and NPY/AgRP/GABA neurons, (ii) NDN is a anorexigenic peptide acting probably via the activation of ARC POMC/CART neurons, and (iii) NDN signaling contributes to the leptin-melanocortin pathway. While the identity of the uncharacterized endozepine metabotropic receptor remains to be fully established, we can postulate that the ARC ACBD7 producing neuron represents a significant relay between leptin and the melanocortin signaling pathway.

## Conclusion

**Figure 1:**
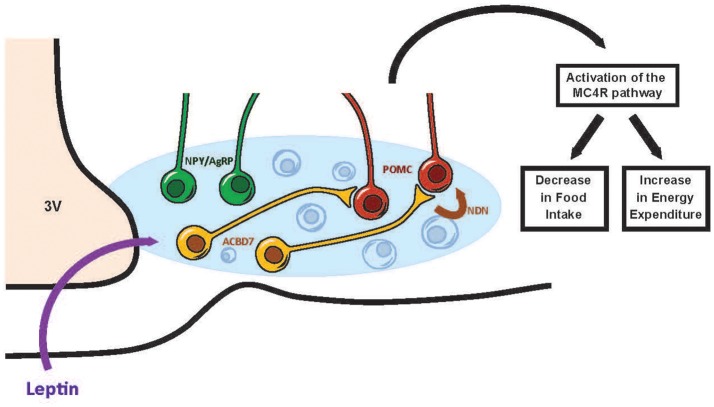
Scheme tentatively summarizing the involvement of ACBD7 and its related product NDN in the effects of leptin in the ARC. According to this scheme, ACBD7-producing neurons would release NDN in response to an increase of leptin (Leptin) concentrations and stimulate the activity of POMC neurons. Activation of POMC neurons of the ARC would lead to the activation of the melanocortin signaling pathway and subsequently to a decrease in food intake and an increase in energy expenditure.

This review has focused on the recent discoveries regarding the hypothalamic leptin signaling pathway and on potential ARC anorectic neurochemical candidates interfacing leptin of the melanocortin system. Up until recently, it was though that leptin action on ARC POMC/CART and NPY/AgRP/GABA neurons was essentially mediated through the leptin receptors (LepR) found on those neurons (Elias et al., 1999; Balthasar et al., 2004; Zhang and Scarpace, 2006). However, recent data have indicated that the leptin effect on the melanocortin system can be relayed by an uncharacterized class of ARC neurons that are distinct from POMC/CART and NPY/AgRP/GABA neurons (Balthasar et al., 2004; van de Wall et al., 2008; Hill et al., 2010; Vong et al., 2011). In this context, the identification of ARC neuromediators other than those released by the POMC/CART and NPY/AgRP/GABA neurons that could relay the catabolic message of leptin via the melanocortin system, appeared justified in our understanding of the pathophysiology of obesity. We identified ARC ACBD7 and its anorexigenic maturation product NDN as playing a role in energy homeostasis. NDN acts mainly by stimulating the melanocortin signaling pathway while NDN signaling disruption blunts the anorexigenic effects of leptin (Figure 1). Future investigations to further examine the involvement of ACBD7 production by ARC Lepr-producing neurons in the hypothalamic leptin signaling pathway appear warranted.

## Author contributions

DL contributed to manuscript preparation and manuscript definition of intellectual content. DR also contributed to manuscript preparation and followed by manuscript editing and revision.

### Conflict of interest statement

The authors declare that the research was conducted in the absence of any commercial or financial relationships that could be construed as a potential conflict of interest.
